# Contrasting population genetic structures of freshwater eels (*Anguilla marmorata, A. bicolor, A. mossambica*) from Madagascar

**DOI:** 10.1111/jfb.70612

**Published:** 2026-08-18

**Authors:** Giada Speranza, Estelle Razafindranaivo, Denis Miranto Andriamahefa, Meelis Tambets, Marc Sztatecsny, Fanomezana Ratsoavina, Zafimahery Rakotomalala, Julia M. I. Barth, Michael Matschiner, Chrysoula Gubili, Robert Jehle, Robert Schabetsberger

**Affiliations:** ^1^ School of Science, Engineering and Environment University of Salford Salford UK; ^2^ Mention Zoologie et Biodiversité Animale Faculté des Sciences, Université d'Antananarivo Antananarivo Madagascar; ^3^ Estonian Marine Institute University of Tartu Tartu Estonia; ^4^ Environment Agency Austria Vienna Austria; ^5^ Zoological Institute, Department of Environmental Science University of Basel Basel Switzerland; ^6^ Centre for Ecological and Evolutionary Synthesis (CEES), Department of Biosciences University of Oslo Oslo Norway; ^7^ Ludwig Maximillians University Munich Planegg‐Martinsried Germany; ^8^ Fisheries Research Institute Hellenic Agricultural Organization‐DIMITRA Nea Peramos Kavala Greece; ^9^ Department of Environment and Biodiversity University of Salzburg Salzburg Austria

**Keywords:** Indian Ocean, spawning areas, sympatry, tropical eels

## Abstract

Freshwater eels (genus *Anguilla*) are of major biological and commercial interest due to their complex catadromous life cycle and as a high‐value fishery resource, but the biology of tropical species is still poorly understood. This study investigates the genetic integrity, diversity and population structure of three species of tropical eels (*Anguilla marmorata, A. bicolor* and *A. mossambica*) in the western Indian Ocean, based on partial nucleotide sequences of the mtDNA control region (*n* = 107 samples) and the nuclear GTH2b gene (*n* = 91 samples) for individuals collected from two freshwater systems in northern Madagascar. No first‐generation hybrids between the studied species were found, despite frequent heterozygosities at the nuclear locus. For the mtDNA control region, all species were characterized by high haplotype diversity (ranging between 0.992 and 0.996) and nucleotide diversity (ranging between 0.035 and 0.059), patterns that are consistent with a historical population expansion during the late Pleistocene as inferred from demographic modelling. In line with previous studies, we confirmed distinct mtDNA haplogroups in *A. marmorata* and *A. bicolor* which can be attributed to the partial and/or past separation of populations across the Indian Ocean, whereas the western Indian Ocean endemic *A. mossambica* revealed no structure. Our findings align with existing evidence for spawning areas in the western Indian Ocean for all three species, with *A. marmorata* partially separated from eastern Indian Ocean populations and *A. bicolor* showing higher connectivity. Our findings will aid in the future management of local anguillid stocks.

## 1 INTRODUCTION

The genus *Anguilla* (freshwater eels) is the only extant representative of the teleost fish family Anguiliidae and comprises 19 species and subspecies distributed in and around the Indian Ocean, the western and southern Pacific and the northern Atlantic (e.g., Arai, 2020). Anguillids are renowned for a catadromous life history whereby mature individuals migrate from freshwater and estuarine habitats to oceanic spawning areas, from where larvae known as leptocephali are transported by currents back towards continental waters (e.g., Schmidt, 1922; Tesch, 1977; Aida et al., 2003). This leads to a distinctive structure of their populations, and for the temperate species (*Anguilla anguilla, A. japonica* and *A. rostrata*), a panmictic breeding strategy results in a single, broadly homogenous gene pool with high levels of genetic variation (Avise et al., 1986; Mank & Avise, 2003; Pujolar, Jacobsen, Als, Frydenberg, Munch, et al., 2014; Gong et al., 2019; Enbody et al., 2021). Anguillids are also of high economic importance, with the import and export of eels and their products having a global value of several billion USD annually (FAO, 2024), and sharp declines of the widely harvested temperate species led to a shift in commercial interest towards eels from tropical regions (Arai, 2014; Dekker & Casselman, 2014; Limburg & Waldman, 2009; Wirth & Bernatchez, 2003). A full picture of population genetic structures for all tropical species, including those with potentially multiple spawning areas, is still outstanding and thus remains a key research and management question (Aoyama, 2023; Faulks et al., 2025; Righton et al., 2021).

A remarkable feature of the genus *Anguilla* is that several species regularly share spawning areas, which can facilitate interspecific hybridisation. The best‐studied example is represented by the Atlantic *A. anguilla* and *A. rostrata* spawning in the Sargasso Sea, with first‐generation hybrids predominantly found in Iceland while low levels of introgression are detectable throughout the continental range of both species (Albert et al., 2006; Pujolar, Jacobsen, Als, Frydenberg, Magnussen, et al., 2014; Tengstedt et al., 2025; Wielgoss et al., 2014). As a further example, frequencies of first‐ and second‐generation hybrids across three species (*A. marmorata*, *A. megastoma* and *A. obscura*) exceeded 20% for individuals caught on the island of Gaua in the southwestern Pacific, with hybrid breakdown and cytonuclear incompatibilities in introgressed individuals ensuring the cohesion of species (Barth et al., 2020; Schabetsberger et al., 2015). To what extent hybridisation is pervasive in other tropical regions where several *Anguilla* species meet has yet to be documented.

The western Indian Ocean is characterized by a complex pattern of surface currents flowing in easterly, westerly and southerly directions (Biastoch et al., 2024; Marsh & van Sebille, 2021), and is home to four species of freshwater eels, all of which are distributed across mainland Africa as well as Madagascar (*A. bengalensis, A. bicolor, A*. *marmorata* and *A. mossambica*; e.g., Arai, 2020). Investigations of their population genetic structures have so far focused on single species, either to infer their ocean‐wide diversification (e.g., Gagnaire et al., 2009, 2011; Ishikawa et al., 2004; Minegishi et al., 2008, 2012) or their local differentiation (Elmy et al., 2024; Frankowski et al., 2020). Comparative genetic inferences across several species sampled in shared freshwater habitats would provide additional information about their local natural history (see e.g. Robinet et al., 2008 for evidence on possible migration routes based on local species compositions) but are so far largely lacking.

The present study aims to provide further insights into the genetic structure of three freshwater eels (*A. marmorata, A. bicolor* and *A. mossambica*) sampled from northern Madagascar. More specifically, this study employs information drawn from nuclear and mitochondrial DNA sequences to (i) screen the sampled individuals for evidence of between‐species hybridisation and (ii) document their population structures and standing levels of genetic variation to infer their local natural histories as well as historic effective population sizes in a biogeographic context. By doing so, this study provides important information for the future conservation management of anguillids in the western Indian Ocean.

## 2 METHODS

### 2.1 Ethics statement

The study was conducted in accordance with all relevant guidelines and policies. The field work in Madagascar was conducted under the following permits: 036/22/MEDD/SG/DGGE/DAPRNE/SCBE.Re and 044/23/MEDD/SG/DGGE/DAPRNE/SCBE.Re (sampling, issued by the Secrétariat général, Direction générale de la gouvernance environnementale, Direction des aires protégées, des ressources naturelles renouvelables et des ecosystems); 7/2022/MEDD/SG/DREDD.ANLJ/CEF.512, 211/22/MEDD/SG/DREDD/ANLJ/SRF and 55/23/MEDD/SG/DREDD ALM/CEF AZK (transport, issued by the Secrétariat Général, Direction Générale de l'Environnement et du Developpement durable Analanjirofo, Cantonennement de l'Environnement et des Forêts Maroantsetra, the Secrétariat Général, Direction Regionale Analanjirofo, Service Regionale des Forêts and the Secrétariat Général, Direction Generale de l'Environnement et du Developpement durable Alaotra Mangoro, Cantonennement de l'Environnement et des Forets Ambatondrazaka) and 122 N‐EA06/MG22 and 196 N‐EA05/MG23 (export, issued by the Secrétariat Général, Direction Générale de la Gouvernance Environnementale, Direction des Aires Protégées, des Ressources Naturelles Renouvelables et des Ecosystems). The laboratory work (DNA sample processing) was approved by the ethics committee of the University of Salford.

### 2.2 Sample collection

Samples of all three species (*n =* 48 for *A. marmorata, n* = 32 for *A. bicolor, n* = 1 for *A. mossambica*) were collected from streams on the Masoala Peninsula (localities: Anaovandrano, Iketry, Fampotabe and Antalavia) in May 2022. Additional samples of *A. mossambica* (*n* = 37) were collected in April 2023 from the Maningory River (locality Ankarongana; Figure 1). Eels were captured by local fishermen with hook and line and by the research team using fykenets and electrofishing (Grassl ELT62II backpack unit). Captured individuals were anaesthetized in a clove oil bath until motionless. Total length, as well as the distances from the lower jaw to the anus and to the origin of the dorsal fin, were measured to the nearest millimetre. All individuals were weighed, and morphologically assigned to species based on body proportions, colouration and dentition of the upper jaw in the field following Ege (1939; for details on all captured individuals, including those for which no DNA are available, see Table S1). A small clip of the pectoral fin was taken and preserved in absolute ethanol for genetic analyses, after which the eels were immediately released.

**FIGURE 1:**
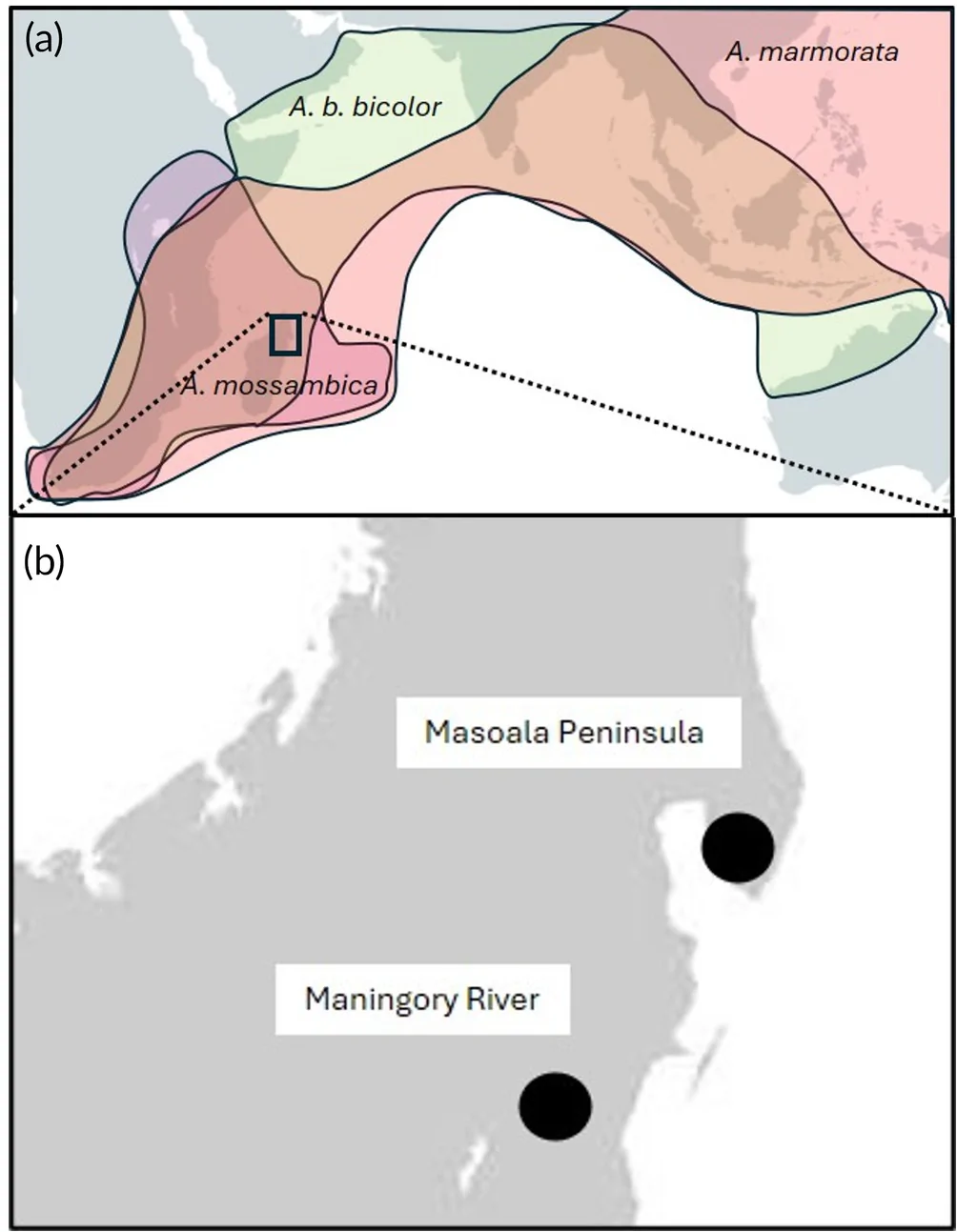
(a) Schematic distributions of the three study species (*A. marmorata, A. mossambica, A. bicolor*) in and around the Indian Ocean, with northern Madagascar highlighted; (b) Outline map of northern Madagascar, showing the two sampling locations; samples of *A. marmorata* and *A. bicolor* were collected on the Masoala Peninsula, and samples of *A*. *mossambica* were mainly collected in the Maningory River.

### 2.3 Laboratory work

Genomic DNA was extracted using Qiagen DNEasy Blood & Tissue extraction kits following the manufacturer's instructions and calibrated to a concentration of approximately 10 ng/μl. Following a previous study by Schabetsberger et al. (2015) and Gubili et al. (2019) on five *Anguilla* species in the south western Pacific, we sequenced one mitochondrial DNA locus (partial control region, CR) using the primers MTF (5′‐GACTTTAACTTCCATCCTCAACTCCC‐3′) and MTR (5′‐GGGAACTTGAAATAGGAGCCAAATGCCAG‐3′) to amplify a 527 bp fragment, and one nuclear locus (gonadotropin II‐beta subunit, GTH2b) using the primers GTH2bF (5′ TCAATCATCTTACATCTGCAGC‐3′) and GTH2bR (5′‐TAACCTTATTCTGTCAAGTGCG‐3′) to amplify a 280 bp fragment. Amplifications of the mtDNA CR locus were carried out in 20 μL reaction volumes with 2 μL of 10x reaction buffer NH_4_, 0.6 μL of 50 mM MgCl_2_, 2 μL of 1 mM deoxyribonucleotide triphosphate (dNTP), 0.4 μL of each primer and 0.5 μL of Taq (all reagents Bioline) and 14.5 μL polymerase chain reaction (PCR) water, using the following temperature profile: 5 min at 94°C, followed by 35 cycles of 30 s at 94°C, 30 s at 58°C and 30 s at 72°C, concluded with 5 min at 72°C. Amplifications for the GTH2bF locus used the same 20 μL reaction mix and general thermal profile, with an annealing temperature of 52°C. Each PCR reaction was tested for successful amplification using 1.5% agarose gels prior to commercial Sanger sequencing (Macrogen, the Netherlands).

### 2.4 Population genetic analyses

All sequences were checked using ProSeq3 (Filatov, 2009), and aligned and trimmed on MEGA11 (Tamura et al., 2021). After trimming, the final size of the sequences was 450 bp for the CR and 266 bp for the GHT2b gene. The quality of sequences was checked on Geneious Prime 2025 (https://www.geneious.com), retaining sequences with >80% classified as High Quality. The resulting sequences were entered the BLAST function of the National Center for Biotechnology Information (NCBI) nucleotide database for species assignment (https://www.ncbi.nlm.nih.gov/), recording the percentage of nucleotide matching to already deposited sequences. For the CR sequence, we considered the result with the highest identification value, whereas for the GHT2b sequence, all results with 100% query cover as well as results at >98% identification with a query cover ranging between 95% and 99% were considered. For the nuclear sequence, *A. mossambica* presented heterozygous nucleotides in two individuals at position 99 (C/T), and in eight individuals at position 141 (A/G). Thus, nuclear‐phased haplotypes were estimated using Bayesian methods on PHASE v2.1.1 (Stephens & Donnelly, 2003).

Number of haplotypes (*H*), haplotype diversity (*h*), number of polymorphic sites and nucleotide diversity (*π*) were calculated using DnaSP 6.0 (Librado & Rozas, 2009). Molecular diversity indices were calculated on ARLEQUIN v3.5.2.2 (Excoffier & Lischer, 2010) using the pairwise difference option. ARLEQUIN was also used to compute neutrality tests, assessing Tajima's *D* (Tajima, 1989) and Fu's *F*_s_ (Fu & Li, 1993) with 1000 simulated samples, allowing to investigate the presence of non‐random changes in population sizes, levels of genetic drift and the presence of bottleneck events (Excoffier & Lischer, 2010). Phylogenetic trees for each locus were constructed using Geneious Prime 2025.

For the phylogenetic network analyses, the CR sequences documented in this study were integrated into existing datasets of the three species collected from the GenBank database (see Table S2; note that sample numbers were renamed for the network to increase simplicity). For *A. mossambica*, all sequences were used. For *A. bicolor*, only sequences of *A. bicolor bicolor* were considered to avoid taxonomic complications and for *A. marmorata*, only sequences from the Indian Ocean and the Indo‐Pacific Seas were included due to its large range and the large number of haplotypes available. For each species‐specific alignment, ambiguously aligned regions were removed using BMGE v1.12 (Block Mapping and Gathering with Entropy; Criscuolo & Gribaldo, 2010). This reduced the alignments by 74 (483 to 409) for *A. marmorata*, 17 (472 to 455) for *A. bicolor bicolor* and 6 (456 to 450) nucleotides for *A. mossambica*. A maximum‐likelihood phylogeny was inferred using IQ‐TREE v1.6.12 (Nguyen et al., 2015), with ModelFinder used to identify the best‐fitting DNA substitution model, which was chosen according to BIC (*A. marmorata* TIM3+F+I+G4; *A. bicolor bicolor* TPM2u+F+R3; *A. mossambica* HKY+F+G4). The phylogenetic networks were constructed using Hapsolutely v0.2.3 (Vences et al., 2024) based on Fitch distances, considering either both transitions and transversions or transversions only.

Mutation models for the CR gene were constructed using BEAUTi and BEAST v1.8.4 (Drummond & Rambaut, 2007). For *A. mossambica* all samples except MAD2/1 were used (due to its highly differentiated genotype, see below). The HKY nucleotide substitution model and the strict clock model were applied, using a uniform distribution for the clock rate with an initial value of 2.5^−8^, an upper value 2.51^−8^ and a lower value 2.49^−8^, at a chain length of 10^8^. Bayesian skyline plot (BSP) models were constructed on Tracer v1.7 (Rambaut, 2018) using the evolutionary models built on BEAST. Due to a lower level of polymorphism in the nuclear locus, we conducted this analysis with the mtDNA gene (CR locus) only.

## 3 RESULTS

### 3.1 Species identification and evidence for hybridisation

After accounting for PCR and sequencing failures, the sample sizes were *n* = 40, 32 and 35 for the mtDNA CR locus of *A. marmorata, A. bicolor* and *A. mossambica*, respectively (GenBank Accession Numbers: PZ698420‐698526), and *n* = 38, 19 and 34 for the GHT2b locus (GenBank Accession Numbers: PZ718737‐PZ718827), notably with 41.1% amplification failures for *A. bicolor*. The results from BLAST (NCBI) revealed a concordance between morphological and mitochondrial as well as nuclear genetic species identification for all samples (where available). For the mtDNA CR locus, 12 (30%) *A. marmorata*, 3 (9.4%) *A. bicolor* and no *A. mossambica* had 100% BLAST matches with previously documented haplotypes; all 40 *A. marmorata*, 26 (81%) *A. bicolor* and 17 (47.2%) of *A. mossambica* individuals found matches at >98.0% similarity, with one *A. mossambica* (MAD2/1) standing out with a low similarity of 91.5% at no other closer matches. For the GTH2b locus, the similarity was 99.3% for all *A. marmorata* except two individuals (99% and 100% similarities, respectively); no comparable sequences are available for *A. bicolor* and A. *mossambica*. The GHT2b sequence diagnostically identified *A. marmorata*, with identical sequences between some *A. bicolor* and *A. mossambica* (data not shown); it also contained a heterozygote nucleotide in ten (29.4%) of *A. mossambica* individuals, and in two (10.5%) of *A. bicolor* individuals.

### 3.2 Genetic diversity and population structure

The DNA polymorphism analyses (Table 1) revealed consistently high haplotype diversities for the mtDNA CR locus for all three species (*h* between 0.988 and 0.997), with nucleotide diversities ranging between 0.032 and 0.059; for the nuclear GHT2b locus, the corresponding values are 0.102–0.883 (*h*) and 0.001–0.015 (*π*). The total number of CR haplotypes (*H*) was 32 for *A. marmorata*, 30 for *A. bicolor* and 33 for *A. mossambica*; for the GHT2b sequence, 2, 7 and 13 genotypes were revealed for *A. marmorata, A. bicolor* and *A. mossambica*, respectively. Accordingly, the numbers of polymorphic sites (*s*) ranged between 76 and 121 for the mtDNA CR locus and between 2 and 13 for the GHT2b locus. Tajima's *D* and Fu's *F* resulted in negative values for all three species for the mtDNA CR locus (values ranging between −0.588 and −1.840 and between −18.284 and −10.615, respectively, with the latter being statistically significant). For the GHT2b sequence, the respective values ranged from −1.090 to 0.931 and from −3.490 to 0.843, respectively, without any statistical significance (Table 1).

**TABLE 1:** Sequence polymorphism analyses for *Anguilla marmorata*, *A. bicolor bicolor* and *A. mossambica* of one mitochondrial (CR, control region) and one nuclear sequence (GHT2b). *π*, nucleotide diversity; *D*, Tajima's D; *F*, Fu's F; *h*, haplotype diversity; *H*, number of haplotypes; *n*, number of sequences; *s*, number of polymorphic sites.

Species	*n*	*H*	*h*	*π*	*s*	*D*	*F*
CR locus:							
*A. marmorata*	40	32	0.988 (± 0.008)	0.032 (± 0.005)	76	−0.922 (*p =* 0.192)	−11.330 (*p* = 0.006)
*A. bicolor*	32	30	0.996 (± 0.009)	0.059 (± 0.029)	121	−0.588 (*p =* 0.299)	−10.615 (*p =* 0.006)
*A. mossambica*	35	33	0.997 (±0.008)	0.035 (± 0.003)	116	−1.840 (*p =* 0.018)	−18.284 (*p* < 0.001)
GHT2b locus:							
*A. marmorata*	38	2	0.102 (± 0.065)	0.001 (± 0.001)	2	−1.090 (*p =* 0.175)	0.257 (*p =* 0.315)
*A. bicolor*	19	7	0.830 (± 0.054)	0.015 (± 0.008)	13	0.931 (*p =* 0.857)	0.843 (*p =* 0.680)
*A. mossambica*	34	13	0.8830 (± 0.020)	0.009 (± 0.006)	12	−0.214 (*p =* 0.461)	−3.490 (*p =* 0.083)

The phylogenetic tree of the mtDNA CR locus revealed an unambiguous distinction of the three species, presenting *A. marmorata* and *A. bicolor* as sister species to *A. mossambica* (Figure S1). Considering transversions only (Figure 2) as well as transitions and transversions (Figure S2), the Indian Ocean mtDNA CR haplotype networks for *A. marmorata* revealed haplogroups which partly combined eastern and western Indian Ocean populations. The haplotypes revealed by the present study appear to be panmictic with existing haplotypes from Reunion, forming their own haplogroup with however a small number of samples overlapping with a haplogroup predominantly from Indonesia (Ambon). The *A. bicolor* haplotypes, on the other hand, were rather uniformly distributed across two main haplogroups which accordingly comprised both eastern and western Indian Ocean populations (Figure 2, Figure S2). *Anguilla mossambica* is confined to the western Indian Ocean where it appeared to form one coherent haplogroup except for a single outlier individual (Figure S2, Table S2).

**FIGURE 2:**
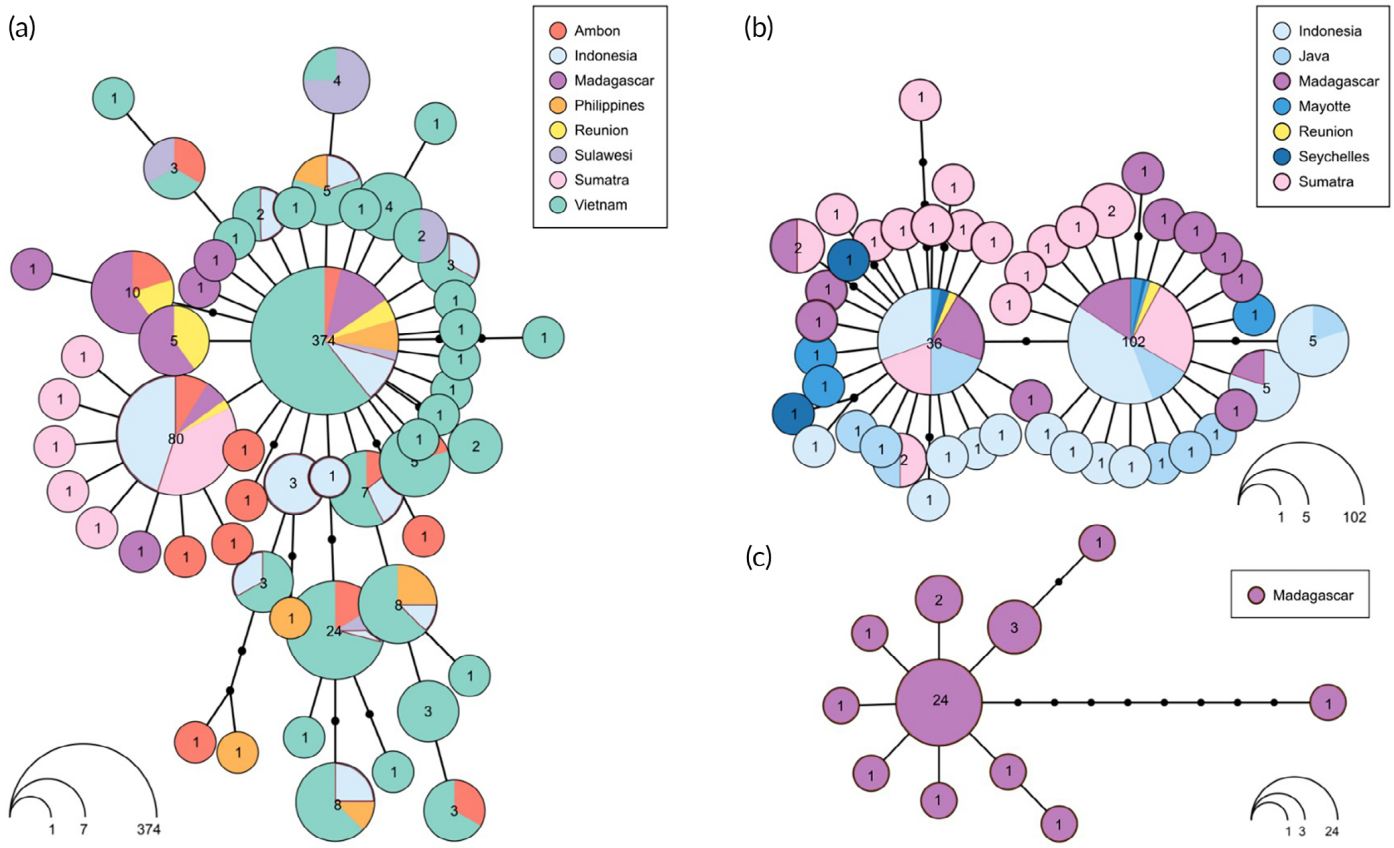
Mitochondrial DNA (control region) haplotype networks for *Anguilla marmorata* (a), *A. bicolor bicolor* (b) and *A. mossambica* (c) in the Indian Ocean, considering transitions only. For sample details, see Table S2.

The BSPs revealed a historic increase in effective population size for all species. *A. marmorata* was subjected to a slight decline between 200,000 and 150,000 years ago (BP) followed by a rapid expansion plateauing around 100,000 BP. *Anguilla bicolor* shows a steady increase that started 900,000 BP, plateauing around 200,000 BP. *A. mossambica* shows a rapid increase in population size that started around 200,000 BP, plateauing around 50,000 BP (Figure 3).

**FIGURE 3:**
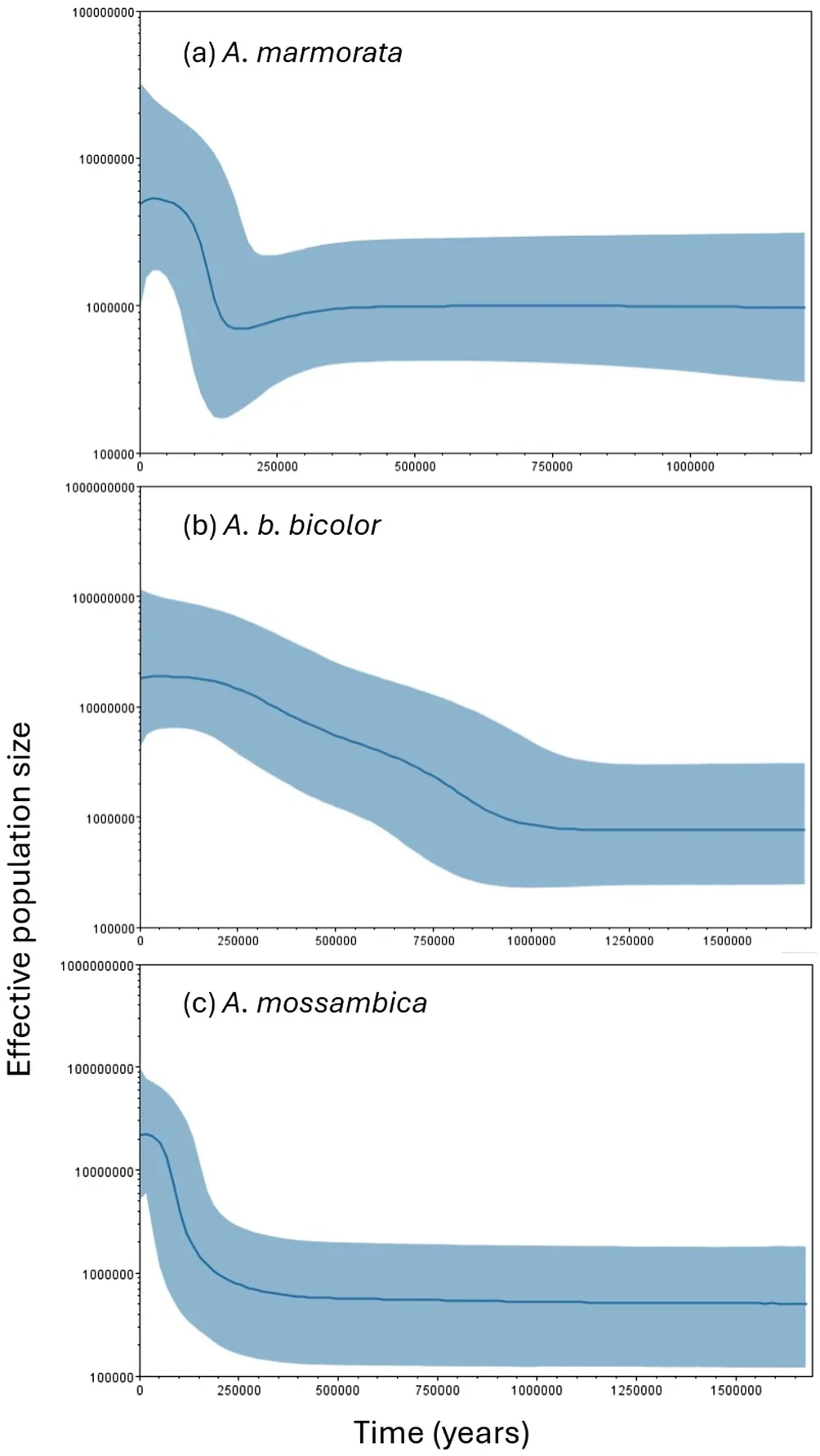
Bayesian Skyline Plots based on partial control region mtDNA sequences for *Anguilla marmorata* (a), *A. bicolor* (b) and *A. mossambica* (c) to model their effective population sizes over time.

## 4 DISCUSSION

This study focused on the population structure of *A. marmorata, A. bicolor* and *A. mossambica* sampled from freshwater habitats in Madagascar, employing the same genetic markers (sequences of the partial mtDNA control region and a nuclear sequence) which were previously used for five *Anguilla* species in the southwestern Pacific (including *A. marmorata*; Schabetsberger et al., 2015; Gubili et al., 2019). The amplification of the partial mtDNA control region using the MTF/MTR primer set continued to consistently yield in sequence data. The amplification of the nuclear GHT2b locus, however, was unsuccessful for 13/32 (41.1%) of *A. bicolor* samples, and the obtained sequences were not fully diagnostic to separate *A. bicolor* from *A. mossambica* (Speranza, 2025). We thus retained the nuclear sequence information to provide partial insights into possible between‐species hybridisation but conducted the inferences of population structures and demographic histories on mtDNA information alone.

### 4.1 Genetic evidence for hybridisation

Based on full concordances between morphological, mitochondrial and (where applicable) nuclear genetic species designations, we found no evidence for hybrid individuals. This finding is in stark contrast to, for example, the island of Gaua in the southwestern Pacific, where >20% of individuals across three species were found to be of hybrid origin, providing evidence for interspecific matings in a common spawning area (Barth et al., 2020; an initial study was based on the same set of genetic markers as the present investigation; Schabetsberger et al., 2015). For the present study site in the western Indian Ocean, a hydrodynamic model showed that the local distribution of leptocephali and glass eels is compatible with a spawning area east of Madagascar that is shared between the study species (Pous et al., 2010; but see also Robinet et al., 2003). If this is indeed the case, then the lack of first‐generation hybrids observed herein could be due to a lower propensity of the studied species assemblage to undergo interspecific matings, reduced fitness of individuals resulting from interspecific matings or due to temporal segregation of reproduction (as suggested by Pous et al., 2010). Notably, however, we also found a high number (*n =* 12) of individuals that were heterozygote in the nuclear GHT2b gene, which in the South Pacific supported the *A. marmorata*—*A. megastoma* hybrid status of individuals bearing discordances between mtDNA and morphology (Schabetsberger et al., 2015). Given their high proportions in the absence of evidence for first‐ or second‐generation hybrids and the lack of comparative sequence data from non‐overlapping parts of the global *A. mossambica* and *A. bicolor* ranges, we tentatively interpret such genotypes as within‐species polymorphisms rather than evidence for ongoing between‐species introgression. Given the inability of the nuclear sequences to diagnostically separate *A. mossambica* and *A. bicolor*, they could however be indicative of interbreeding earlier in the species' evolutionary histories.

### 4.2 Genetic diversity and population demography

The levels of similarity of the mtDNA haplotypes revealed by the present study with sequences available on GenBank generally reflect the amount of existing information for each study species. For example, this is the first quantitative investigation of *A. mossambica* based on the mtDNA CR locus (the two already available haplotypes stem from single specimens; Ishikawa et al., 2004; Minegishi et al., 2005), and all haplotypes presented in this study have not been previously recorded.

For *A. marmorata*, we revealed high CR haplotype diversity and a high number of polymorphic sites, similar to other studies conducted in the Indian Ocean and the western Pacific based on the same locus (Gubili et al., 2019; Minegishi et al., 2008; Nguyen et al., 2022; Schabetsberger et al., 2015) as well as based on other loci (Arai & Taha, 2021; Marini et al., 2024). The same is the case for *A. bicolor*, with high haplotype diversity as previously documented in South East Asia (Marini et al., 2021). Despite being restricted to the western Indian Ocean and possibly linked to higher demographic stability, *A. mossambica* possessed a number of haplotypes and polymorphic sites, which were comparable to the more widespread *A. marmorata* and *A. bicolor*, with the lowest haplotype diversity and a more pronounced signature of population expansion based on Tajima's *D* and Fu's *F*. The latter test statistics have a generally higher statistical power to detect recent population expansions and were significant for all three study species, whereas a Tajima's *D* is further indicative of positive selection and was not significant for *A. marmorata* and *A. bicolor* (species with more partitioned gene pools and occurring in the western and eastern Indian Ocean; a similar pattern was also found by Gubili et al., 2019 for Pacific eels).

Despite differential levels of significance, the generally negative Tajima's *D* and Fu's *F* are in line with the modelled effective population sizes, for *A. marmorata* and *A. mossambica* revealing an increase primarily in the last 150,000 years which coincides with sea level rises during the late Pleistocene (for other examples from the southwestern Pacific see Visram et al., 2010; Hoareau et al., 2012). Such a pattern of population increase was also observed for four eel species, including *A. marmorata*, in the southeastern Pacific (Gubili et al., 2019), as well as for other *A. mossambica* populations from the western Indian Ocean (Frankowski et al., 2020). The effective population size of *A. bicolor*, on the other hand, appeared to increase more steadily over a longer time frame of one million years, similar to populations from South East Asia (*A. bicolor pacifica*, Marini et al., 2021) and further corroborated by population rises over shorter timescales in the Indo‐Pacific (Minegishi et al., 2012; spanning 14,000 years) and East Africa (Elmy et al., 2024; spanning 250,000 years). The difference between *A. bicolor* compared to the other two study species could be due to its population split in the Indian Ocean approximately 140,000 years ago, followed by secondary contact thereafter (Minegishi et al., 2012). It however should be borne in mind that the inferences are based on a single locus with limited variability and should thus be treated with caution.

### 4.3 Local population genetic structure, with implications for spawning migrations

A main finding of the present study is that the investigated eel species differ in their population structure, representing a single (*A. mossambica*) or several (*A. bicolor* and *A. marmorata*) mtDNA haplogroups despite their syntopy in northern Madagascar. Two non‐mutually exclusive explanations can account for this observation. Firstly, the three species underwent differential evolutionary histories, with the existence of several haplogroups indicative of past population subdivisions. Secondly, distinct haplogroups within given species can arise through the current use of separated spawning areas followed by sympatry during the freshwater phase.

*A. marmorata* is currently the only anguillid which clearly lacks panmixis, leading to the assumption of multiple spawning areas across its range (for a summary see Faulks et al., 2025). It is distributed across the Indian as well as Pacific Ocean, with the emergence of the Sunda and Sahul shelves during the Pleistocene having potentially led to a temporary separation of populations on either side of the Indo‐Australian archipelago (as observed in a range of taxa, e.g., Hobbs et al., 2009). In addition to genetic groups partitioned within the Pacific Ocean, *A. marmorata* is further separated into eastern and western populations in the Indian Ocean, with suspected spawning areas west of Sumatra and east of Madagascar, respectively (Gagnaire et al., 2009, 2011; Ishikawa et al., 2004; Minegishi et al., 2008; Watanabe et al., 2008). Our finding of a dominant haplogroup in *A. marmorata* in addition to more distant individuals which are part of otherwise Asian populations conforms to previous studies in the western Indian Ocean (Ishikawa et al., 2004; Minegishi et al., 2008). This pattern has previously been interpreted as secondary contact of distinct eastern and western populations through occasional unidirectional larval dispersal westward along the South Equatorial Current (Gagnaire et al., 2009), with a lack of population isolation further evidenced through admixed individuals and low levels of nuclear divergence (Gagnaire et al., 2011; Minegishi et al., 2008). A western Indian Ocean spawning ground which separates the local *A. marmorata* populations from the eastern Indian Ocean is also in line with otolith growth rates in leptocephali that suggest a shared environment (Robinet et al., 2008), as well as a local hydrodynamic model based on the distribution of larvae (Pous et al., 2010; leptocephali of *A. marmorata* were already found northeast of Madagascar during the DANA expedition in 1929/30; Jespersen, 1942).

For *A. bicolor*, the individuals from Madagascar were rather equally distributed across two previously documented distinct haplogroups without pronounced geographic signatures across the Indian Ocean (Minegishi et al., 2012; Arai & Taha, 2021; but see also Marini et al., 2021), leading to a less compact clustering of individuals compared to the majority of the *A. marmorata* individuals from Madagascar (Figure 2). The two haplogroups are best explained by ancestral population separation through the rise of the Mascarene ridge, followed by the mixing of populations through east–west migration loops facilitated by the South Equatorial and Equatorial Counter Currents, the long larval period of *A. bicolor*, and its almost year‐long breeding (Minegishi et al., 2012). A clear subdivision into two main haplogroups was however not detected for populations from Tanzania (Elmy et al., 2024). Additonally considering the evidence for separate migration routes for leptocephali in the western Indian Ocean based on otolith growth rates (Robinet et al., 2008), this also gives rise to the possible exististence of multiple spawning grounds or partially allochronic breeding cohorts (see also Robinet et al., 2003, who, based on glass eels sampled in a fishery of Réunion Island, provide evidence for an *A. bicolor* spawning ground which is separate from *A. marmorata* and *A. mossambica*). Future studies on *A. bicolor* should use multi‐locus nuclear markers to investigate its subtle local population genetic differentiation (as e.g., conducted for *A. marmorata*, Gagnaire et al., 2011).

In contrast to our other study species (*A. marmorata* and *A. bicolor*), the western Indian Ocean endemic *A. mossambica* revealed no population substructure, in line with findings by Frankowski et al. (2020) across a wider area and thus providing evidence for a single panmictic spawning area. During the DANA expedition (Jespersen, 1942), leptocephali of *A. mossambica* were collected in the same area as *A. marmorata*, but at however different developmental stages and thus suggesting a potential use of the same spawning area at different times (see also Pous et al., 2010).

The conservation management of *Anguilla* species in the western Indian Ocean is currently hampered by incomplete information on migration, spawning areas and population structure. The main finding of the present study is that we found no indication of between‐species hybridisation despite our data conforming to previous evidence of at least partly shared spawning areas. We also show that, despite being sampled in nearby freshwater environments in Madagascar, the three study species were characterized by markedly differing haplotype diversities, likely reflecting their range‐wide evolutionary histories and confirming existing evidence from previous investigations (Ishikawa et al., 2004; Minegishi et al., 2008). These findings have important implications for the management of these populations in view of ensuring their sustainable viability. Future studies should consider a wider systematic sampling of eels along the western Indian Ocean to investigate the possibility of more localized hybrid occurrences (in the North Atlantic, for example, hybrids are only found on Iceland, and in the South Pacific, a hybridisation hotspot is represented by Gaua; Barth et al., 2020; Tengstedt et al., 2025). In view of the increasing harvest demand and thus need to manage the existing stocks, this would also facilitate a better picture of the spatial distribution of the documented *A. marmorata* and *A. bicolor* haplogroups across Madagascar and the African continent.

## AUTHOR CONTRIBUTIONS

Giada Speranza: Conducted laboratory work and data analysis; co‐wrote and reviewed the manuscript. Estelle Razafindranaivo: Coordinated and conducted field sampling and data collection. Denis Miranto Andriamahefa: Conducted field sampling and data collection. Meelis Tambets: Conducted field sampling and data collection. Marc Sztatecsny: Conducted field sampling and data collection. Fanomezana Ratsoavina: Coordinated the international collaboration between the Universities of Antananarivo and Salzburg; obtained the research permits. Zafimahery Rakotomalala: Coordinated the international collaboration between the Universities of Antananarivo and Salzburg; obtained the research permits. Julia M. I. Barth: Contributed to the data analysis, reviewed the manuscript, funding acquisition (co‐PI). Michael Matschiner: Helped with the data analysis, reviewed the manuscript, funding acquisition (co‐PI). Chrysoula Gubili: Supervised the study, coordinated and contributed to the data analysis, reviewed the manuscript, funding acquisition (co‐PI). Robert Jehle: Supervised the study, co‐wrote and reviewed the manuscript, funding acquisition (co‐PI). Robert Schabetsberger: Conceived and supervised the study, coordinated international collaboration, coordinated and conducted field sampling and data collection, contributed to data interpretation, reviewed the manuscript, funding acquisition (PI). All authors approved the final version of the manuscript.

## FUNDING INFORMATION

This study was funded by the Austrian Science Fund (FWF, Grant number P34091‐B; Principle Investigator R. Schabetsberger). Part of the laboratory work was additionally funded by the University of Salford. Chrysoula Gubili was supported by a Royal Society Wolfson Visiting Fellowship (RSWVF\R2\232003).

## CONFLICT OF INTEREST STATEMENT

The authors declare no conflicts of interest.

## Supporting information


**Figure S1.** Phylogenetic tree for *Anguilla marmorata*, *A. bicolor bicolor* and *A. mossambica* based on partial mtDNA control region sequences for samples collected in Madagascar. For sample details, see Table S2.

**Figure S2.** Mitochondrial DNA (control region) haplotype networks for *Anguilla marmorata* (A), *A. bicolor bicolor* (B) and *A. mossambica* (C) in the Indian Ocean, considering transitions and transversions. For sample details, see Table S2.

**Table S1.** List of individuals recorded during field work and their measurements. Y: sampling year; L: sampling location (A – Anovandrano, collected 2022; M – Maningory River, collected 2023); W: weight (kg); T: total length (cm); A: snout‐anus length (cm); D: snout‐dorsal fin length (cm); G: gill opening (cm); M: mouth (cm); Eh (horizontal eye diameter, mm); Ev (vertical eye diameter, mm); P: left pectoral fin length (mm).

**Table S2.** Sample designations used for the network analyses based on partial control region mtDNA sequences for *Anguilla marmorata, A. bicolor bicolor* and *A. mossambica*. GenBank accession numbers reflect sample numbers detailed in Table S1, with sample designators unified for Figure 2.

## Data Availability

All newly created sequences are available through GenBank (CR locus: Accession Numbers PZ698420‐698526; GHT2b locus: Accession Numbers PZ718737‐PZ718827).

## References

[jfb70612-bib-0056] Aida, K. , Tsukamoto, K. , & Yamauchi, K. (Eds.). (2003). Eel biology. Springer.

[jfb70612-bib-0001] Albert, V. , Jónsson, B. , & Bernatchez, L. (2006). Natural hybrids in Atlantic eels (*Anguilla anguilla, A. rostrata*): Evidence for successful reproduction and fluctuating abundance in space and time. Molecular Ecology, 15, 1903–1916. 10.1111/j.1365-294X.2006.02917.x 16689906

[jfb70612-bib-0002] Aoyama, J. (2023). Population structure and speciation. In K. Tsukamoto , M. Kuroki , & S. Watanabe (Eds.), Eel Science. Fisheries Science Series (pp. 23–36). Springer.

[jfb70612-bib-0003] Arai, T. (2014). Do we protect freshwater eels or do we drive them to extinction? SpringerPlus, 3, 1–10. 10.1186/2193-1801-3-534 PMC4174548 25279325

[jfb70612-bib-0004] Arai, T. (2020). Ecology and evolution of migration in the freshwater eels of the genus *Anguilla* Schrank, 1798. Heliyon, 6(10), e05176. 10.1016/j.heliyon.2020.e05176 PMC7553983 33083623

[jfb70612-bib-0005] Arai, T. , & Taha, H. (2021). Contrasting patterns of genetic population structure in tropical freshwater eels of genus *Anguilla* in the Indo‐Pacific. Heliyon, 7(5), e05176. 10.1016/j.heliyon.2021.e07097 PMC8144006 34041412

[jfb70612-bib-0058] Avise, J. C. , Helfman, G. S. , Saunders, N. C. , & Stanton, H. S. (1986). Mitochondrial DNA differentiation in North Atlantic eels: Population genetic consequences of an unusual life history pattern. Proceedings of the National Academy of Sciences, 83, 4350–4354. 10.1073/pnas.83.12.4350 PMC323730 16593712

[jfb70612-bib-0006] Barth, J. M. I. , Gubili, C. , Matschiner, M. , Torresen, O. K. , Watanabe, S. , Egger, B. , Han, Y. S. , Feunteun, E. , Sommaruga, R. , Jehle, R. , & Schabetsberger, R. (2020). Stable species boundaries despite ten million years of hybridization in tropical eels. Nature Communications, 11(1), 1433. 10.1038/s41467-020-15099-x PMC7080837 32188850

[jfb70612-bib-0007] Biastoch, A. , Rühs, S. , Ivanciu, I. , Schwarzkopf, F. u. , Veitch, J. , Reason, C. , Zorita, E. , Tim, N. , Hünicke, B. , Vafeidis, A. t. , Santamaria‐Aguilar, S. , Kupfer, S. , & Soltau, F. (2024). The Agulhas current system as an important driver for oceanic and terrestrial climate. In Ecological Studies (Vol. 248). Springer. 10.1007/978-3-031-10948-5_8

[jfb70612-bib-0008] Criscuolo, A. , & Gribaldo, S. (2010). BMGE (block mapping and gathering with entropy): A new software for selection of phylogenetic informative regions from multiple sequence alignments. BMC Evolutionary Biology, 10, 210. 10.1186/1471-2148-10-210 PMC3017758 20626897

[jfb70612-bib-0009] Dekker, W. , & Casselman, J. M. (2014). The 2003 Québec declaration of concern about eel declines—11 years later: Are eels climbing back up the slippery slope? Fisheries, 39(12), 613–614.

[jfb70612-bib-0010] Drummond, A. , & Rambaut, A. (2007). BEAST: Bayesian evolutionary analysis by sampling trees. BMC Evolutionary Biology, 7, 214. 10.1186/1471-2148-7-214 PMC2247476 17996036

[jfb70612-bib-0011] Ege, V. (1939). A revision of the genus *Anguilla* Shaw, a systematic, phylogenetic and geographical study. Dana Repport, Carlsberg Foundation, 16, 1–256.

[jfb70612-bib-0012] Elmy, F. I. , Gaspare, L. , Mfilinge, P. L. , & Lusana, J. L. (2024). Genetic structure and demographic history of *Anguilla bicolor* from Tanzania based on mitochondrial cytochrome oxidase I sequence: Implications for effective management and conservation strategies. Aquatic Conservation: Marine and Freshwater Ecosystems, 34(7), e4225.

[jfb70612-bib-0060] Enbody, E. D. , Pettersson, M. E. , Grace Sprehn, C. , & Andersson, L. (2021). Ecological adaptation in European eels is based on phenotypic plasticity. Proceedings of the National Academy of Sciences, 118, e2022620118. 10.1073/pnas.2022620118 PMC7848574 33479174

[jfb70612-bib-0013] Excoffier, L. , & Lischer, H. E. L. (2010). Arlequin suite ver 3.5: A new series of programs to perform population genetics analyses under Linux and windows. Molecular Ecology Resources, 10(3), 564–567. 10.1111/j.1755-0998.2010.02847.x 21565059

[jfb70612-bib-0014] FAO . (2024). Global aquatic trade statistics. In In: Fisheries and aquaculture. Food and Agriculture Organisation. https://www.fao.org/fishery/en/collection/global_commodity_prod.

[jfb70612-bib-0015] Faulks, L. , Daryani, A. , & Hakoyama, H. (2025). Panmixia in *Anguilla* eels: A meta‐analysis. Fish and Fisheries, 26, 895–908.

[jfb70612-bib-0016] Filatov, D. A. (2009). Processing and population genetic analysis of multigenic datasets with ProSeq3 software. Bioinformatics, 25(23), 3189–3190. 10.1093/bioinformatics/btp572 PMC2778335 19797407

[jfb70612-bib-0017] Frankowski, J. , Lübke, K. , Coke, M. , & Weyl, O. L. (2020). Genetic variability and demographic history of *Anguilla mossambica* (Peters, 1852) from continental Africa and Madagascar. Journal of Fish Biology, 96(5), 1251–1259. 10.1111/jfb.14220 31777080

[jfb70612-bib-0018] Fu, Y. X. , & Li, W. H. (1993). Statistical tests of neutrality of mutations. Genetics, 133(3), 693–709. 10.1093/genetics/133.3.693 PMC1205353 8454210

[jfb70612-bib-0020] Gagnaire, P. A. , Minegishi, Y. , Zenboudji, S. , Valade, P. , Aoyama, J. , & Berrebi, P. (2011). Within‐population structure highlighted by differential introgression across semipermeable barriers to gene flow in *Anguilla marmorata* . Evolution, 65(12), 3413–3427. 10.1111/j.1558-5646.2011.01404.x 22133215

[jfb70612-bib-0019] Gagnaire, P.‐A. , Minegishi, Y. , Aoyama, J. , Reveillac, E. , Robinet, T. , Bosc, P. , Tsukamoto, K. , Feunteun, E. , & Berrebi, P. (2009). Ocean currents drive secondary contact between *Anguilla marmorata* populations in the Indian Ocean. Marine Ecology Progress Series, 379, 267–278.

[jfb70612-bib-0059] Gong, X. , Davenport, E. R. , Wang, D. , & Clark, A. G. (2019). Lack of spatial and temporal genetic structure of Japanese eel (*Anguilla japonica*) populations. Conservation Genetics, 20, 467–475.

[jfb70612-bib-0021] Gubili, C. , Schabetsberger, R. , Poellabauer, C. , Bates, B. , Wagstaff, R. M. , Woodward, L. M. , Sichrowsky, U. , Scheck, A. , Boseto, D. T. , Feunteun, E. , Acou, A. , & Jehle, R. (2019). High genetic diversity and lack of pronounced population structure in five species of sympatric Pacific eels. Fisheries Management and Ecology, 26(1), 31–41. 10.1111/fme.12287 PMC7116419 33244193

[jfb70612-bib-0022] Hoareau, T. B. , Boissin, E. , & Berrebi, P. (2012). Evolutionary history of a widespread Indo‐Pacific goby: The role of Pleistocene sea‐level changes on demographic contraction/expansion dynamics. Molecular Phylogenetics and Evolution, 62, 566–572. 10.1016/j.ympev.2011.10.004 22037473

[jfb70612-bib-0023] Hobbs, J.‐P. A. , Frisch, A. J. , Allen, G. R. , & Van Herwerden, L. (2009). Marine hybrid hotspot at Indo‐Pacific biogeographic border. Biology Letters, 5, 258–261. 10.1098/rsbl.2008.0561 PMC2665801 19126528

[jfb70612-bib-0024] Ishikawa, S. , Tsukamoto, K. , & Nishida, M. (2004). Genetic evidence for multiple geographic populations of the giant mottled eel *Anguilla marmorata* in the Pacific and Indian oceans. Ichthyological Research, 51(4), 343–353.

[jfb70612-bib-0025] Jespersen, P. (1942). Indo‐Pacific Leptocephalids of the genus Anguilla‐systematic and biological studies. Carlsberg Foundation.

[jfb70612-bib-0026] Librado, P. , & Rozas, J. (2009). DnaSP v5: A software for comprehensive analysis of DNA polymorphism data. Bioinformatics, 25(11), 1451–1452. 10.1093/bioinformatics/btp187 19346325

[jfb70612-bib-0027] Limburg, K. E. , & Waldman, J. R. (2009). Dramatic declines in North Atlantic diadromous fishes. Bioscience, 59(11), 955–965. 10.1525/bio.2009.59.11.7n

[jfb70612-bib-0057] Mank, J. E. , & Avise, J. C. (2003). Microsatellite variation and differentiation in Atlantic eels. Journal of Heredity, 94, 310–314. 10.1093/jhered/esg062 12920102

[jfb70612-bib-0061] Marini, M. , Pedrosa‐Gerasmio, I. R. , Santos, M. D. , Romana‐Eguia, M. R. R. , Nguyen, B. T. , & Shibuno, T. (2024). High genetic diversity in anguillid glass eels across three southeast Asian countries revealed through mitochondrial DNA D‐loop sequences, with notes on population structure. Ecological Genetics and Genomics, 32, 100276.

[jfb70612-bib-0028] Marini, M. , Pedrosa‐Gerasmio, I. R. , Santos, M. D. , Shibuno, T. , Daryani, A. , Romana‐Eguia, M. R. R. , & Wibowo, A. (2021). Genetic diversity, population structure and demographic history of the tropical eel *Anguilla bicolor pacifica* in Southeast Asia using mitochondrial DNA control region sequences. Global Ecology and Conservation, 26, e01493.

[jfb70612-bib-0029] Marsh, R. , & van Sebille, E. (2021). Chapter 6 ‐ the tropical oceans, interannual climate variability, and ecosystem adaptation. In R. Marsh & E. van Sebille (Eds.), Ocean currents (pp. 189–239). Elsevier.

[jfb70612-bib-0030] Minegishi, Y. , Aoyama, J. , & Tsukamoto, K. (2008). Multiple population structure of the giant mottled eel, Anguilla Marmorata. Molecular Ecology, 17(13), 3109–3122. 10.1111/j.1365-294X.2008.03822.x 18522690

[jfb70612-bib-0031] Minegishi, Y. , Aoyama, Y. , Inoue, J. G. , Miya, M. , Nishida, M. , & Tsukamoto, K. (2005). Molecular phylogeny and evolution of the freshwater eels genus *Anguilla* based on the whole mitochondrial genome sequences. Molecular Phylogenetics and Evolution, 34(1), 134–146. 10.1016/j.ympev.2004.09.003 15579387

[jfb70612-bib-0032] Minegishi, Y. , Gagnaire, P.‐A. , Aoyama, J. , Bosc, P. , Feunteun, E. , Tsukamoto, K. , & Berrebi, P. (2012). Present and past genetic connectivity of the Indo‐Pacific tropical eel *Anguilla bicolor* . Journal of Biogeography, 39(2), 408–420.

[jfb70612-bib-0033] Nguyen, A. T. , Dao, H. T. , Quang, H. T. , Hagihara, S. , Lokman, P. M. , & Damsteegt, E. L. (2022). Genetic diversity and population structure of the giant mottled eel, *Anguilla marmorata* Quoy & Gaimard, 1824 in Central Vietnam. Fishes, 7(5), 286.

[jfb70612-bib-0034] Nguyen, L.‐T. , Schmidt, H. A. , von Haeseler, A. , & Minh, B. Q. (2015). IQ‐TREE: A fast and effective stochastic algorithm for estimating maximum‐likelihood phylogenies. Molecular Biology and Evolution, 32, 268–274. 10.1093/molbev/msu300 PMC4271533 25371430

[jfb70612-bib-0035] Pous, S. , Feunteun, E. , & Ellien, C. (2010). Investigation of tropical eel spawning area in the South‐Western Indian Ocean: Influence of the oceanic circulation. Progress in Oceanography, 86(3), 396–413.

[jfb70612-bib-0036] Pujolar, J. M. , Jacobsen, M. W. , Als, T. D. , Frydenberg, J. , Magnussen, E. , Jónsson, B. , Jiang, X. , Cheng, L. , Bekkevold, D. , Maes, G. E. , Bernatchez, L. , & Hansen, M. M. (2014). Assessing patterns of hybridization between North Atlantic eels using diagnostic single‐nucleotide polymorphisms. Heredity, 112(6), 627–637. 10.1038/hdy.2013.145 PMC4023445 24424165

[jfb70612-bib-0037] Pujolar, J. M. , Jacobsen, M. W. , Als, T. D. , Frydenberg, J. , Munch, K. , Jónsson, B. , Jin, J. B. , Cheng, L. , Maes, G. E. , Bernatchez, L. , & Hansen, M. M. (2014). Genome‐wide single‐generation signatures of local selection in the panmictic European eel. Molecular Ecology, 23, 2514–2528. 10.1111/mec.12753 24750353

[jfb70612-bib-0039] Rambaut, A. (2018). Figtree ver 1.4.4. Institute of Evolutionary Biology, University of Edinburgh.

[jfb70612-bib-0040] Righton, D. , Piper, A. , Aarestrup, K. , Amilhat, E. , Belpaire, C. , Casselman, J. , Castonguay, M. , Díaz, E. , Dörner, H. , Faliex, E. , Feunteun, E. , Fukuda, N. , Hanel, R. , Hanzen, C. , Jellyman, D. , Kaifu, K. , McCarthy, K. , Miller, M. J. , Pratt, T. , … Gollock, M. (2021). Important questions to progress science and sustainable management of anguillid eels. Fish and Fisheries, 22, 762–788.

[jfb70612-bib-0041] Robinet, T. , Lecomte‐Finiger, R. , Escoubeyrou, K. , & Feunteun, E. (2003). Tropical eels *Anguilla* spp. recruiting to Réunion island in the Indian Oceans: Taxonomy, patterns of recruitment and early life histories. Marine Ecology Progress Series, 259, 263–272.

[jfb70612-bib-0042] Robinet, T. , Réveillac, E. , Kuroki, M. , Aoyama, J. , Tsukamoto, K. , Rabenevanana, M. W. , Valade, P. , Gagnaire, P.‐A. , Berrebi, P. , & Feunteun, E. (2008). New clues for freshwater eels (*Anguilla* spp.) migration routes to eastern Madagascar and surrounding islands. Marine Biology, 154, 453–463.

[jfb70612-bib-0043] Schabetsberger, R. , Okland, F. , Kalfatak, D. , Sichrowsky, U. , Tambets, M. , Aarestrup, K. , Gubili, C. , Sarginson, J. , Boufana, B. , Jehle, R. , Dall'Olmo, G. , Miller, M. J. , Scheck, A. , Kaiser, R. , & Quartly, G. (2015). Genetic and migratory evidence for sympatric spawning of tropical Pacific eels from Vanuatu. Marine Ecology Progress Series, 521, 171–187.

[jfb70612-bib-0044] Schmidt, J. (1922). On the life history of the water‐eels (Anguillidae), with special reference to the development of the *Anguilla vulgaris* and *Anguilla japonica* . Meddelelser Fra Kommissionen for Danmarks Fiskeri‐ Og Havundersøgelser, 6, 1–258.

[jfb70612-bib-0045] Speranza, G. (2025). Genetic diversity and population structure of *Anguilla marmorata*, *Anguilla bicolor* and *Anguilla mossambica* in the Indian Ocean. Unpublished MSc by Research thesis. University of Salford.

[jfb70612-bib-0046] Stephens, M. , & Donnelly, P. (2003). A comparison of Bayesian methods for haplotype reconstruction from population genotype data. American Journal of Human Genetics, 73(5), 1162–1169. 10.1086/379378 PMC1180495 14574645

[jfb70612-bib-0047] Tajima, F. (1989). Statistical method for testing the neutral mutation hypothesis by DNA polymorphism. Genetics, 123(3), 585–595. 10.1093/genetics/123.3.585 PMC1203831 2513255

[jfb70612-bib-0048] Tamura, K. , Stecher, G. , & Kumar, S. (2021). MEGA11: Molecular evolutionary genetics analysis version 11. Molecular Biology and Evolution, 38(7), 3022–3027. 10.1093/molbev/msab120 PMC8233496 33892491

[jfb70612-bib-0049] Tengstedt, A. N. B. , Liu, S. , Jacobsen, M. W. , Ulmo‐Diaz, G. , Jónsson, B. , Pujolar, J. M. , & Hansen, M. M. (2025). Genomic footprints of hybridisation in North Atlantic eels (*Anguilla anguilla* and *A. rostrata*). Molecular Ecology, 34, e17664. 10.1111/mec.17664 PMC12617339 39878237

[jfb70612-bib-0055] Tesch, F.‐W. (1977). The eels: Biology and management of anguillid eels. Chapman and Hall.

[jfb70612-bib-0050] Vences, M. , Patmanidis, S. , Schmidt, J.‐C. , Matschiner, M. , Aurélien, M. , & Renner, S. (2024). Hapsolutely: A user‐friendly tool integrating haplotype phasing, network construction and haploweb calculation. Bioinformatics Advances, 4, vbae083. 10.1093/bioadv/vbae083 PMC11184345 38895561

[jfb70612-bib-0051] Visram, S. , Yang, M. C. , Pillay, R. M. , Said, S. , Henriksson, O. , Grahn, M. , & Chen, C. A. (2010). Genetic connectivity and historical demography of the blue barred parrotfish (*Scarus ghobban*) in the western Indian Ocean. Marine Biology, 157, 1475–1487.

[jfb70612-bib-0052] Watanabe, S. , Aoyama, J. , Miller, M. J. , Ishikawa, S. , Feunteun, E. , & Tsukamoto, K. (2008). Evidence of population structure in the giant mottled eel, *Anguilla marmorata*, using total number of vertebrae. Copeia, 2008, 680–688.

[jfb70612-bib-0053] Wielgoss, S. , Gilabert, A. , Meyer, A. , & Wirth, T. (2014). Introgressive hybridization and latitudinal admixture clines in North Atlantic eels. BMC Evolutionary Biology, 14, 61. 10.1186/1471-2148-14-61 PMC3986858 24674242

[jfb70612-bib-0054] Wirth, T. , & Bernatchez, L. (2003). Decline of North Atlantic eels: A fatal synergy? Proceedings of the Royal Society London B, 270, 681–688. 10.1098/rspb.2002.2301 PMC1691294 12713741

