# Supplementary material for: Contrasting population genetic structures of freshwater eels (Anguilla marmorata, A. bicolor, A. mossambica) from Madagascar
Source: J Fish Biol. 2026 Aug 18;109(4):2409–18. doi: 10.1111/jfb.70612 (PMC13675488; doi:10.1111/jfb.70612)
Supplement: Supplementary file 1 [file JFB-109-2409-s002.docx]

| **Sample** | **L** | **W** | **T** | **A** | **D** | **G** | **M** | **Eh** | **Ev** | **P** | **Colour** | |
| --- | --- | --- | --- | --- | --- | --- | --- | --- | --- | --- | --- | --- |
| ***Anguilla marmorata*** | | | | | | | | | | | |  |
| MAD1 | A | 0.6 | 65 | 29 | 18 | 10 | 3.5 | 8.9 | 10.9 | 36 | marbled | |
| MAD2 | A | 0.1 | 40 | 17.5 | 10.5 | 5.4 | 1.9 | 4 | 4.6 | 20 | marbled | |
| MAD4 | A | < 0.1 | 34.5 | 14.5 | 8.5 | 4.8 | 1.4 | 3.6 | 3.9 | 15.2 | marbled | |
| MAD5 | A | < 0.1 | 21.4 | 8.7 | 5.3 | 2.3 | 0.8 | 1.9 | 1.9 | 6.9 | marbled | |
| MAD6 | A | 0.3 | 51 | 22.4 | 13.8 | 7.2 | 2.7 | 5 | 5.5 | 23.7 | marbled | |
| MAD7 | A | 0.3 | 46.6 | 19.8 | 12.1 | 6.4 | 2.1 | 5.5 | 5.5 | 21.3 | marbled | |
| MAD8 | A | 0.8 | 66 | 29 | 16.4 | 9.5 | 3.2 | 9.4 | 9.6 | 30.4 | marbled | |
| MAD9 | A | 1.83 | 95.4 | 45 | 28 | 15.5 | 5.6 | 10 | 9.4 | 56 | marbled | |
| MAD10 | A | 0.55 | 60.4 | 27.8 | 16.6 | 9.7 | 3.3 | 6.3 | 6.4 | 30.6 | marbled | |
| MAD12 | A | 0.46 | 67.8 | 30 | 18.4 | 10 | 3 | 8.8 | 7.4 | 40 | marbled | |
| MAD21 | A | 0.22 | 48.3 | 21.3 | 12.8 | 7 | 2.3 | 4.1 | 4.5 | 23.7 | marbled | |
| MAD22 | A | 0.97 | 73.7 | 33.7 | 19 | 10.6 | 3.4 | 7.8 | 6.7 | 38.1 | marbled | |
| MAD23 | A | 1.18 | 73.3 | 34 | 19.8 | 10.3 | 3.5 | 8.7 | 7.4 | 38.6 | marbled | |
| MAD24 | A | 0.87 | 70.1 | 31.3 | 19.3 | 10.8 | 3.8 | 7.9 | 6.4 | 35.5 | marbled | |
| MAD25 | A | 1.19 | 76.5 | 36.5 | 21.4 | 12.6 | 4.4 | 8.9 | 8 | 44.8 | marbled | |
| MAD39 | A | 0.11 | 37.1 | 15.7 | 9.1 | 5.2 | 1.7 | 3.5 | 4.1 | 15.7 | marbled | |
| MAD46 | A | 4.79 | 118 | 53.7 | 32.3 | 16.7 | 5.6 | 11.2 | 10.9 | 68.2 | marbled | |
| MAD47 | A | 1.14 | 71.8 | 32.7 | 19.1 | 10.6 | 3.4 | 7.3 | 6.9 | 33.6 | marbled | |
| MAD48 | A | 0.76 | 65.7 | 28.8 | 16.9 | 9.5 | 2.9 | 6.9 | 6.2 | 28.6 | marbled | |
| MAD49 | A | 1.01 | 72.7 | 33.8 | 21.1 | 11.9 | 4.2 | 8 | 7.3 | 42.1 | marbled | |
| MAD50 | A | 0.81 | 67.2 | 35.5 | 21.4 | 10.4 | 3.1 | 6.7 | 6.3 | 35.2 | marbled | |
| MAD51 | A | 0.61 | 62.3 | 28.2 | 17.5 | 9.2 | 3 | 7.6 | 7 | 34.1 | marbled | |
| MAD52 | A | 1.23 | 86.2 | 35.6 | 21.1 | 11.6 | 4.1 | 7.4 | 7 | 43.2 | marbled | |
| MAD53 | A | 0.36 | 52.3 | 22.6 | 13.5 | 7.6 | 2.4 | 6.3 | 5.1 | 24.7 | marbled | |
| MADUP2 | A | <0,1 | 24.5 | 10 | 5.7 | 3.3 | 0.8 | 1.5 | 1.6 | 9 | marbled | |
| MADUP3 | A | 0.3 | 48.6 | 21.1 | 13 | 7 | 2.2 | 3.5 | 3.5 | 22 | marbled | |
| MADUP4 | A | 0.6 | 63.7 | 27.5 | 16.3 | 9 | 2.9 | 5 | 5 | 32 | marbled | |
| MADUP5 | A | 1.2 | 76 | 33.4 | 19.5 | 10.3 | 3.5 | 5 | 5 | 42 | marbled | |
| MADUP6 | A | 0.6 | 60.1 | 25.7 | 15.2 | 8 | 2.6 | 4 | 4 | 27 | marbled | |
| MADUP7 | A | 0.5 | 56.8 | 24.7 | 14.6 | 8.2 | 2.6 | 4.5 | 4.5 | 28 | marbled | |
| MADUP8 | A | 0.3 | 51.7 | 22.9 | 13.6 | 6.8 | 2.8 | 5.1 | 5.1 | 27 | marbled | |
| MADUP9 | A | 0.2 | 44.2 | 18.4 | 10.8 | 6.2 | 1.7 | 4 | 4 | 19 | marbled | |
| MADUP10 | A | <0,1 | 20.6 | 8.6 | 4.8 | 2.7 | 0.8 | 1.5 | 1.5 | 6 | marbled | |
| MADUP11 | A | 1.1 | 73 | 35 | 19.6 | 10.6 | 3.4 | 6.5 | 6.5 | 38 | marbled | |
| MADUP12 | A | 0.6 | 61.9 | 26.6 | 16 | 8.5 | 2.7 | 5 | 5 | 31 | marbled | |
| MADUP13 | A | 1.8 | 85.5 | 37.2 | 22.5 | 11 | 3.8 | 8.5 | 8.5 | 47 | marbled | |
| MADUP14 | A | 2.3 | 96.8 | 43.1 | 27 | 14.8 | 4.6 | 9 | 8 | 48 | marbled | |
| MADUP15 | A | 2 | 91.2 | 39.2 | 23.1 | 11.8 | 3.9 | 9 | 8 | 49 | marbled | |
| MADUP | A | 0.12 | 46 | 19.1 | 11.1 | 5.9 | 1.9 | 4 | 4.5 | 21 | marbled | |
| MADUP16 | A | 0.9 | 70.1 | 30 | 17.2 | 9.4 | 2.7 | 6 | 6 | 26 | marbled | |
| MADUP17 | A | 1.7 | 86.3 | 39.2 | 23.4 | 12.6 | 3.9 | 10 | 8 | 49 | marbled | |
| MADUP18 | A | 1 | 72.8 | 31.5 | 18.6 | 9.9 | 3.3 | 9 | 8 | 44 | marbled | |
| MADUP19 | A | 2.7 | 95.6 | 41.5 | 24.8 | 13.1 | 4.2 | 9 | 8 | 52 | marbled | |
| MAD60 | A | 0.75 | 67.5 | 27.4 | 17.8 | 10.1 | 3.3 | 6.4 | 6.7 | 34.4 | marbled | |
| MAD61 | A | 0.32 | 78.2 | 24.6 | 15.2 | 8.1 | 2.8 | 5.8 | 5.5 | 29.9 | marbled | |
| MAD64 | A | 1 | 71.5 | 34.5 | 17.8 | 11.2 | 3.7 | 8.2 | 8.2 | 41.6 | marbled | |
| MAD65 | A | 0.1 | 41.7 | 18.1 | 11.3 | 6.2 | 1.8 | 4.6 | 4.4 | 21.5 | marbled | |
| MAD66 | A | 0.42 | 58.3 | 26.6 | 15.2 | 7.9 | 2.8 | 4.8 | 4.7 | 29.9 | marbled | |
| MAD67 | A | 0.3 | 37.8 | 21.3 | 12.7 | 6.8 | 2.2 | 4.6 | 4.8 | 23.3 | marbled | |
| ***Anguilla bicolor bicolor*** | | | | | | | | | | | |  |
| MAD11 | A | 0.3 | 60 | 27.4 | 27.2 | 8.7 | 2.5 | 6 | 6.3 | 23.7 | plain | |
| MAD13 | A | 0.25 | 51.3 | 22 | 21.5 | 7.2 | 2.2 | 6.1 | 6.1 | 21.4 | plain | |
| MAD14 | A | 0.69 | 75.6 | 32.2 | 32 | 11.5 | 3.8 | 8.3 | 7.5 | 25.1 | plain | |
| MAD15 | A | 0.11 | 28 | 15.7 | 15.1 | 5.1 | 1.4 | 4.4 | 4.1 | 13.7 | plain | |
| MAD16 | A | 0.17 | 46.2 | 20 | 18.7 | 6.7 | 2 | 5.4 | 4.4 | 18.8 | plain | |
| MAD17 | A | 0.37 | 58.6 | 26.6 | 25.7 | 9.8 | 3.2 | 7.2 | 7 | 23.8 | plain | |
| MAD18 | A | 0.05 | 32.4 | 13.8 | 13.3 | 4.5 | 1.3 | 3.7 | 2.8 | 10.2 | plain | |
| MAD19 | A | 0.09 | 39.7 | 16.5 | 16.2 | 6 | 1.6 | 5.7 | 5.3 | 16 | plain | |
| MAD20 | A | 0.22 | 47.4 | 20 | 19.8 | 7 | 2.1 | 5.8 | 5.5 | 18.3 | plain | |
| MAD26 | A | 0.12 | 44.7 | 18.4 | 17.5 | 6.6 | 1.7 | 6.3 | 5.6 | 17.3 | plain | |
| MAD27 | A | 0.14 | 70.4 | 32.4 | 29.3 | 11.2 | 3.7 | 7.7 | 7.4 | 33.9 | plain | |
| MAD28 | A | 0.13 | 44 | 18.7 | 18.5 | 6.5 | 2 | 6.1 | 5.2 | 15.5 | plain | |
| MAD29 | A | 0.15 | 56.2 | 23.8 | 22.3 | 7.9 | 2.6 | 7 | 6.4 | 22.3 | plain | |
| MAD30 | A | 0.13 | 41.2 | 17.5 | 16.5 | 5.9 | 1.9 | 5.3 | 5.1 | 16.1 | plain | |
| MAD31 | A | 0.31 | 60.2 | 29 | 28.3 | 8.7 | 2.9 | 7.1 | 7.1 | 24.7 | plain | |
| MAD32 | A | 0.08 | 36.3 | 15.5 | 14.7 | 4.8 | 1.7 | 6.1 | 3.8 | 11.4 | plain | |
| MAD33 | A | 0.13 | 39.4 | 16.9 | 16.2 | 5.7 | 1.8 | 5.4 | 5 | 13.7 | plain | |
| MAD34 | A | 0.15 | 45.3 | 19.5 | 18.4 | 6.8 | 2 | 5.7 | 4.7 | 18.5 | plain | |
| MAD35 | A | 0.12 | 37.7 | 15.6 | 15.1 | 5 | 1.7 | 4.4 | 4.4 | 14 | plain | |
| MAD36 | A | 0.21 | 46.5 | 19.7 | 18.7 | 6.1 | 1.8 | 7 | 5.8 | 18.6 | plain | |
| MAD37 | A | 0.54 | 68.5 | 30.4 | 28.5 | 10.2 | 3 | 7.2 | 6.9 | 21.9 | plain | |
| MAD38 | A | 0.21 | 50 | 22.4 | 21.3 | 7.4 | 2.3 | 5.9 | 5.3 | 18.3 | plain | |
| MAD40 | A | 0.24 | 51.7 | 21.5 | 20.2 | 7.1 | 2.2 | 5.4 | 5.1 | 19.9 | plain | |
| MAD41 | A | 0.37 | 56.8 | 25.2 | 23.2 | 8.7 | 2.8 | 6.4 | 5.9 | 25.5 | plain | |
| MAD42 | A | 0.13 | 40.4 | 17 | 16.4 | 5.5 | 1.6 | 4.4 | 4.7 | 14.5 | plain | |
| MAD43 | A | 0.13 | 40 | 17 | 16.1 | 5.5 | 1.5 | 4.3 | 4.5 | 15.1 | plain | |
| MAD44 | A | 0.26 | 51.3 | 22.3 | 21.4 | 7.1 | 2 | 6 | 5.8 | 20.5 | plain | |
| MAD45 | A | 0.2 | 47.3 | 19.6 | 18.5 | 6.4 | 1.8 | 5.2 | 5.3 | 17.3 | plain | |
| MAD54 | A | 0.36 | 66.2 | 28 | 25.9 | 8.6 | 2.8 | 7.6 | 6.8 | 27.6 | plain | |
| MAD55 | A | 2.33 | 107.7 | 44.8 | 44 | 13.5 | 4.8 | 10.5 | 10.5 | 45 | plain | |
| MAD56 | A | 0.28 | 72.6 | 32.7 | 32 | 11.8 | 4 | 8.2 | 8 | 36.6 | plain | |
| MAD57 | A | 0.79 | 78.7 | 34.5 | 33.3 | 12.4 | 3.9 | 9.1 | 8.7 | 42.3 | plain | |
| MAD58 | A | 0.57 | 71.8 | 33.1 | 30.6 | 11.3 | 3.8 | 8.2 | 8 | 36.6 | plain | |
| MAD59 | A | 0.37 | 61.8 | 27 | 27.2 | 9.2 | 2.5 | 9.2 | 8.7 | 36 | plain | |
| MAD62 | A | 0.35 | 64.3 | 27 | 27.4 | 9.1 | 3.2 | 7.2 | 7.1 | 27.5 | plain | |
| MAD63 | A | 0.76 | 73.5 | 31.2 | 31.1 | 10.4 | 3.7 | 7.2 | 7.3 | 34.7 | plain | |
| ***Anguilla mossambica*** | | | | | | | | | | | |  |
| MAD3 | A | < 0.1 | 17 | 6.7 | 4.4 | 2.1 | 1.02 | 1.5 | 1.5 | 7.7 | plain | |
| MADUP1 | A | <0.1 | 37 | 15.6 | 9.6 | 5.5 | 1.5 | 3.8 | 3.8 | 19 | plain | |
| MAD2/01 | M | 1.13 | 71 | 31 | 21 | 10.5 | 3.8 | 7.3 | 7 | 41.1 | plain | |
| MAD2/02 | M | 0.7 | 62 | 27 | 19.7 | 9.3 | 3.2 | 6.8 | 6 | 30.5 | plain | |
| MAD2/03 | M | 0.91 | 69.2 | 30 | 20.2 | 9.9 | 3.8 | 8.1 | 7.6 | 38.4 | plain | |
| MAD2/04 | M | 0.94 | 70 | 30.5 | 19.1 | 8.6 | 3.5 | 7.8 | 7.7 | 35.8 | plain | |
| MAD2/05 | M | 0.47 | 55.7 | 25 | 16.6 | 9 | 2.8 | 5.7 | 5.2 | 29.7 | plain | |
| MAD2/06 | M | 0.23 | 43 | 17.4 | 12 | 5.8 | 2 | 3.9 | 3.8 | 20.2 | plain | |
| MAD2/07 | M | 0.46 | 57.2 | 25.7 | 17 | 8.7 | 3.4 | 7.2 | 7.6 | 29.9 | plain | |
| MAD2/08 | M | 0.11 | 34.5 | 14.1 | 9.7 | 4.8 | 1.5 | 4 | 3.9 | 16.7 | plain | |
| MAD2/09 | M | 0.17 | 41.2 | 16.5 | 11.6 | 6.1 | 2.1 | 3.6 | 3.4 | 20.4 | plain | |
| MAD2/10 | M | 0.12 | 36 | 15 | 9.9 | 4.7 | 1.8 | 4 | 4.6 | 17 | plain | |
| MAD2/11 | M | 0.41 | 55.6 | 24.1 | 15.1 | 8.8 | 2.9 | 6.4 | 5.9 | 29.5 | plain | |
| MAD2/12 | M | 0.48 | 54.7 | 24.2 | 15.4 | 8.3 | 2.9 | 6.4 | 5.4 | 28.6 | plain | |
| MAD2/13 | M | 0.29 | 48.5 | 20.8 | 14.1 | 7.5 | 2.6 | 6.5 | 5.6 | 21.8 | plain | |
| MAD2/14 | M | 0.53 | 63.3 | 28.5 | 19.1 | 10 | 3.6 | 8.1 | 6.9 | 32.3 | plain | |
| MAD2/15 | M | 0.75 | 66.6 | 29.5 | 19.4 | 10.6 | 3.7 | 8.7 | 7.5 | 37.6 | plain | |
| MAD2/16 | M | 0.23 | 46.5 | 20.1 | 13.2 | 7.2 | 2.3 | 6.1 | 4.9 | 19.9 | plain | |
| MAD2/17 | M | 0.42 | 55.5 | 24.2 | 15.2 | 8.2 | 3 | 7.5 | 6.4 | 35.8 | plain | |
| MAD2/18 | M | 0.35 | 50.7 | 22 | 14.5 | 7.9 | 2.8 | 5.8 | 5.6 | 28.8 | plain | |
| MAD2/19 | M | 1.05 | 74.6 | 33 | 21.3 | 11.5 | 4.1 | 9 | 8.8 | 38.7 | plain | |
| MAD2/20 | M | 0.22 | 41.7 | 18.2 | 12.4 | 6.6 | 2.3 | 4.8 | 4.5 | 22.5 | plain | |
| MAD2/21 | M | 0.19 | 41.1 | 18 | 11.1 | 6 | 2 | 5.4 | 4.9 | 20.7 | plain | |
| MAD2/22 | M | 0.11 | 36.3 | 15.8 | 9.7 | 5.6 | 2 | 5 | 4.6 | 18 | plain | |
| MAD2/23 | M | 0.14 | 39.3 | 16.4 | 10.3 | 5.5 | 2 | 4.7 | 4.1 | 18.1 | plain | |
| MAD2/24 | M | 0.22 | 45.9 | 18.8 | 11.8 | 6.6 | 2.2 | 5.2 | 4.4 | 22.5 | plain | |
| MAD2/25 | M | 0.37 | 52.3 | 21.5 | 14.4 | 8 | 3 | 6.3 | 5.3 | 26 | plain | |
| MAD2/26 | M | 1.04 | 72.8 | 31.7 | 20.2 | 11 | 3.9 | 7.1 | 7.6 | 39.6 | plain | |
| MAD2/27 | M | 0.05 | 37 | 15.6 | 10 | 5.5 | 1.9 | 4.4 | 4.4 | 17.9 | plain | |
| MAD2/28 | M | 0.07 | 40.3 | 17.2 | 11.1 | 6.1 | 1.9 | 4.6 | 4.1 | 18.7 | plain | |
| MAD2/29 | M | 0.1 | 37.7 | 15.6 | 10.7 | 5.6 | 1.9 | 4.9 | 4.4 | 17.5 | plain | |
| MAD2/30 | M | 0.12 | 36.8 | 15.4 | 10.3 | 5.4 | 1.8 | 4.6 | 4.4 | 18.5 | plain | |
| MAD2/31 | M | 0.39 | 54.4 | 23.2 | 15.5 | 8.5 | 3.8 | 7.2 | 6.3 | 26 | plain | |
| MAD2/32 | M | 0.11 | 38.2 | 16.1 | 10.7 | 5.2 | 1.6 | 4.9 | 4 | 16.2 | plain | |
| MAD2/33 | M | 1.59 | 86.8 | 38.4 | 25.8 | 13.1 | 4.8 | 9.3 | 7.9 | 45.9 | plain | |
| MAD2/34 | M | 1.98 | 91.7 | 40.3 | 26.6 | 14.4 | 4.9 | 9.6 | 9.1 | 44.4 | plain | |
| MAD2/35 | M | 0.67 | 64.7 | 35.6 | 18.7 | 9.8 | 3.7 | 8.8 | 7.8 | 33.2 | plain | |
| MAD2/36 | M | 1.2 | 77.9 | 33.4 | 21.6 | 11.6 | 4 | 9.4 | 8.1 | 41.7 | plain | |
| MAD2/37 | M | 2.05 | 95 | 40 | 25.8 | N/A | N/A | N/A | 11.5 | N/A | plain | |
| MAD2/38 | M | 1.46 | 84 | 37 | 23.5 | N/A | N/A | N/A | 9.6 | N/A | plain | |
