# Supplementary material for: Contrasting population genetic structures of freshwater eels (Anguilla marmorata, A. bicolor, A. mossambica) from Madagascar
Source: J Fish Biol. 2026 Aug 18;109(4):2409–18. doi: 10.1111/jfb.70612 (PMC13675488; doi:10.1111/jfb.70612)
Supplement: Supplementary file 2 [file JFB-109-2409-s004.docx]

| **Accession number** | **Network sample ID** | **Location** | **Reference** | |
| --- | --- | --- | --- | --- |
| ***Anguilla marmorata* (Figure 2)** | | | |  |
| AB279279 - AB279314 | Amb1 - Amb36 | Ambon | Minegishi et al., 2008 | |
| AB279232 - AB279255 | Reu1 - Reu24 | Reunion | Minegishi et al., 2008 | |
| AB279196 - AB279231 | Sum1 - Sum36 | Sumatra | Minegishi et al., 2008 | |
| AB279119 - AB279132 | Sul1 - Sul14 | Sulawesi | Minegishi et al., 2008 | |
| AB279107 - AB279118 | Phi1 - Phi12 | Philippines | Minegishi et al., 2008 | |
| MZ392675 - MZ392694 | Phi13 - Phi32 | Philippines | Marini | |
| MZ392429 - MZ392476 | Indo1 - Indo48 | Indonesia | Marini et al., 2024 | |
| MT327754 - MT327790 | Indo49 – Indo85 | Indonesia | Wibowo | |
| AP007242 | Indo86 | Indonesia | Minegishi et al., 2005 | |
| MZ392744 - MZ392837 | Viet1 - Viet94 | Vietnam | Marini et al., 2024 | |
| OQ111528 - OQ111703 | Viet95 - Viet270 | Vietnam | Nguyen et al., 2022 | |
| MZ367206 - MZ367236 | Viet271 - Viet301 | Vietnam | Tang | |
| AB279256 - AB279278 | Mad1 - Mad23 | Madagascar | Minegishi et al., 2008 | |
| PZ698420 (MAD1) | Mad24 | Madagascar | This study | |
| PZ698421 (MAD2) | Mad25 | Madagascar | This study | |
| PZ698422 (MAD4) | Mad26 | Madagascar | This study | |
| PZ698423 (MAD5) | Mad27 | Madagascar | This study | |
| PZ698424 (MAD6) | Mad28 | Madagascar | This study | |
| PZ698425 (MAD8) | Mad29 | Madagascar | This study | |
| PZ698426 (MAD9) | Mad30 | Madagascar | This study | |
| PZ698427 (MAD10) | Mad31 | Madagascar | This study | |
| PZ698428 (MAD12) | Mad32 | Madagascar | This study | |
| PZ698429 (MAD21) | Mad33 | Madagascar | This study | |
| PZ698430 (MAD22) | Mad34 | Madagascar | This study | |
| PZ698431 (MAD23) | Mad35 | Madagascar | This study | |
| PZ698432 (MAD24) | Mad36 | Madagascar | This study | |
| PZ698433 (MAD25) | Mad37 | Madagascar | This study | |
| PZ698434 (MAD46) | Mad38 | Madagascar | This study | |
| PZ698435 (MAD49) | Mad39 | Madagascar | This study | |
| PZ698436 (MAD50) | Mad40 | Madagascar | This study | |
| PZ698437 (MAD51) | Mad41 | Madagascar | This study | |
| PZ698438 (MAD52) | Mad42 | Madagascar | This study | |
| PZ698439 (MAD53) | Mad43 | Madagascar | This study | |
| PZ698440 (MAD61) | Mad44 | Madagascar | This study | |
| PZ698441 (MAD64) | Mad45 | Madagascar | This study | |
| PZ698442 (MAD65) | Mad46 | Madagascar | This study | |
| PZ698443 (MAD67) | Mad47 | Madagascar | This study | |
| PZ698444 (MADUP2) | Mad48 | Madagascar | This study | |
| PZ698445 (MADUP3) | Mad49 | Madagascar | This study | |
| PZ698446 (MADUP4) | Mad50 | Madagascar | This study | |
| PZ698447 (MADUP5) | Mad51 | Madagascar | This study | |
| PZ698448 (MADUP6) | Mad52 | Madagascar | This study | |
| PZ698449 (MADUP7) | Mad53 | Madagascar | This study | |
| PZ698450 (MADUP8) | Mad54 | Madagascar | This study | |
| PZ698451 (MADUP10) | Mad55 | Madagascar | This study | |
| PZ698452 (MADUP11) | Mad56 | Madagascar | This study | |
| PZ698453 (MADUP12) | Mad57 | Madagascar | This study | |
| PZ698454 (MADUP13) | Mad58 | Madagascar | This study | |
| PZ698455 (MADUP14) | Mad59 | Madagascar | This study | |
| PZ698456 (MADUP15) | Mad60 | Madagascar | This study | |
| PZ698457 (MADUP17) | Mad61 | Madagascar | This study | |
| PZ698458 (MADUP18) | Mad62 | Madagascar | This study | |
| PZ698459 (MADUP19) | Mad63 | Madagascar | This study | |
| ***Anguilla bicolor bicolor* (Figure 3a)** | | | | |
| MN630495 - MN630519 | Java1 – Java25 | Java | Taufiq-Spj et al., 2022 | |
| AB278996 - AB279002 | May1 – May7 | Mayotte | Minegishi et al., 2012 | |
| MZ392496 - MZ392563 | Indo1 - Indo68 | Indonesia | Marini et al., 2024 | |
| AB279012 | Reu1 | Reunion | Minegishi et al., 2012 | |
| AB278993 – AB278995 | Reu2 – Reu4 | Reunion | Minegishi et al., 2012 | |
| AB279006 - AB279010 | Sey1 – Sey5 | Seychelles | Minegishi et al., 2012 | |
| AB278943 - AB030657 | Sum1 - Sum48 | Sumatra | Minegishi et al., 2012 | |
| AB279011 | Mad1 | Madagascar | Minegishi et al., 2012 | |
| AB278990 - AB278992 | Mad2 - Mad4 | Madagascar | Minegishi et al., 2012 | |
| PZ698495 (MAD11) | Mad5 | Madagascar | This study | |
| PZ698496 (MAD13) | Mad6 | Madagascar | This study | |
| PZ698497 (MAD14) | Mad7 | Madagascar | This study | |
| PZ698498 (MAD15) | Mad8 | Madagascar | This study | |
| PZ698499 (MAD16) | Mad9 | Madagascar | This study | |
| PZ698500 (MAD17) | Mad10 | Madagascar | This study | |
| PZ698501 (MAD18) | Mad11 | Madagascar | This study | |
| PZ698502 (MAD19) | Mad12 | Madagascar | This study | |
| PZ698503 (MAD20) | Mad13 | Madagascar | This study | |
| PZ698504 (MAD26) | Mad14 | Madagascar | This study | |
| PZ698505 (MAD27) | Mad15 | Madagascar | This study | |
| PZ698506 (MAD28) | Mad16 | Madagascar | This study | |
| PZ698507 (MAD29) | Mad17 | Madagascar | This study | |
| PZ698508 (MAD30) | Mad18 | Madagascar | This study | |
| PZ698509 (MAD31) | Mad19 | Madagascar | This study | |
| PZ698510 (MAD32) | Mad20 | Madagascar | This study | |
| PZ698511 (MAD33) | Mad21 | Madagascar | This study | |
| PZ698512 (MAD34) | Mad22 | Madagascar | This study | |
| PZ698513 (MAD35) | Mad23 | Madagascar | This study | |
| PZ698514 (MAD37) | Mad24 | Madagascar | This study | |
| PZ698515 (MAD41) | Mad25 | Madagascar | This study | |
| PZ698516 (MAD42) | Mad26 | Madagascar | This study | |
| PZ698517 (MAD43) | Mad27 | Madagascar | This study | |
| PZ698518 (MAD44) | Mad28 | Madagascar | This study | |
| PZ698519 (MAD45) | Mad29 | Madagascar | This study | |
| PZ698520 (MAD54) | Mad30 | Madagascar | This study | |
| PZ698521 (MAD56) | Mad31 | Madagascar | This study | |
| PZ698522 (MAD57A) | Mad32 | Madagascar | This study | |
| PZ698523 (MAD58) | Mad33 | Madagascar | This study | |
| PZ698524 (MAD59) | Mad34 | Madagascar | This study | |
| PZ698525 (MAD62) | Mad35 | Madagascar | This study | |
| PZ698526 (MAD63) | Mad36 | Madagascar | This study | |
| ***Anguilla mossambica* (Figure 3b)** | | | |  |
| AB030659 | Mad1 | Madagascar | Ishikawa et al. | |
| AP007244 | Mad2 | Madagascar | Minegishi et al. | |
| PZ698460 (MAD2/1) | Mad3 | Madagascar | This study | |
| PZ698461 (MAD2/2) | Mad4 | Madagascar | This study | |
| PZ698462 (MAD2/3) | Mad5 | Madagascar | This study | |
| PZ698463 (MAD2/4) | Mad6 | Madagascar | This study | |
| PZ698464 (MAD2/6) | Mad7 | Madagascar | This study | |
| PZ698465 (MAD2/7) | Mad8 | Madagascar | This study | |
| PZ698466 (MAD2/8) | Mad9 | Madagascar | This study | |
| PZ698467 (MAD2/9) | Mad10 | Madagascar | This study | |
| PZ698468 (MAD2/10) | Mad11 | Madagascar | This study | |
| PZ698469 (MAD2/11) | Mad12 | Madagascar | This study | |
| PZ698470 (MAD2/12) | Mad13 | Madagascar | This study | |
| PZ698471 (MAD2/13) | Mad14 | Madagascar | This study | |
| PZ698472 (MAD2/14) | Mad15 | Madagascar | This study | |
| PZ698473 (MAD2/15) | Mad16 | Madagascar | This study | |
| PZ698474 (MAD2/16) | Mad17 | Madagascar | This study | |
| PZ698475 (MAD2/17) | Mad18 | Madagascar | This study | |
| PZ698476 (MAD2/19) | Mad19 | Madagascar | This study | |
| PZ698477 (MAD2/20) | Mad20 | Madagascar | This study | |
| PZ698478 (MAD2/21) | Mad21 | Madagascar | This study | |
| PZ698479 (MAD2/22) | Mad22 | Madagascar | This study | |
| PZ698480 (MAD2/23) | Mad23 | Madagascar | This study | |
| PZ698481 (MAD2/24) | Mad24 | Madagascar | This study | |
| PZ698482 (MAD2/25) | Mad25 | Madagascar | This study | |
| PZ698483 (MAD2/26) | Mad26 | Madagascar | This study | |
| PZ698484 (MAD2/27) | Mad27 | Madagascar | This study | |
| PZ698485 (MAD2/28) | Mad28 | Madagascar | This study | |
| PZ698486 (MAD2/29) | Mad29 | Madagascar | This study | |
| PZ698487 (MAD2/30) | Mad30 | Madagascar | This study | |
| PZ698488 (MAD2/31) | Mad31 | Madagascar | This study | |
| PZ698489 (MAD2/33) | Mad32 | Madagascar | This study | |
| PZ698490 (MAD2/34) | Mad33 | Madagascar | This study | |
| PZ698491 (MAD2/35) | Mad34 | Madagascar | This study | |
| PZ698492 (MAD2/36) | Mad35 | Madagascar | This study | |
| PZ698493 (MAD3) | Mad36 | Madagascar | This study | |
| PZ698494 (MADUP1) | Mad_37 | Madagascar | This study | |
